# A narrative review of spatial multi-omics and organ-on-a-chip technologies for cardio-cerebral-renal crosstalk

**DOI:** 10.3389/fbioe.2026.1749227

**Published:** 2026-03-16

**Authors:** Ke Cheng, Wanyi Liang, Shihong Xiong, Yonghui Liang, Xinyue Wang, Shanshan Yang, Yuxin Wang, Na Gong

**Affiliations:** 1 Department of Nephrology, Tianyou Hospital Affiliated to Wuhan University of Science and Technology, Wuhan, Hubei, China; 2 Wuhan University of Science and Technology, Wuhan, Hubei, China; 3 Medical Examination Center, Hubei Provincial Hospital of Integrated Chinese and Western Medicine, Wuhan, Hubei, China; 4 Hubei University of Chinese Medicine, Wuhan, Hubei, China

**Keywords:** cardio-cerebral-renal axis, inter-organ communication, microfluidic organoids, multi-tissue microphysiological systems, spatial multi-omics

## Abstract

**Background:**

Inter-organ communication within the cardio-cerebral-renal axis orchestrates systemic homeostasis and disease progression. However, conventional methods fail to capture its spatiotemporal complexity; consequently, this creates evidence gaps in dynamic network dissection. Therefore, spatial multi-omics and organ-on-a-chip (OOC) technologies enable unprecedented investigation of these pathways.

**Objective:**

We aimed to evaluate the efficacy and safety of spatial multi-omics and organ-on-a-chip (OOC) technologies (intervention) compared to conventional methods (comparator) in decoding cardio-cerebral-renal crosstalk for systemic disorders (population) on outcomes including sensitivity, resolution, and detection accuracy (outcomes).

**Methods:**

We searched PubMed, Cochrane Library, Embase, Web of Science, and ClinicalTrials.gov from January 2021 to December 2024. Two reviewers independently screened studies for inclusion. We included 34 studies comprising randomized trials, cohort studies, and preclinical validations. We employed narrative synthesis and random-effects meta-analysis where appropriate.

**Results:**

Based on 34 studies involving a substantial number of participants and samples, spatial multi-omics and OOC technologies demonstrated improved sensitivity and better identification of pathogenic networks compared to conventional methods. The findings suggest a consistent trend toward enhanced performance, though heterogeneity across studies was noted.

**Conclusion:**

Building on these findings, we conclude that these technologies establish a novel paradigm for decoding multi-organ communication, revealing molecular mechanisms (e.g., exosomal miRNA regulation) and providing clinically actionable biomarkers. However, evidence certainty is moderate due to heterogeneity, thus supporting future precision medicine applications.

## 1 Introduction

### 1.1 Background

Cardio-cerebral-renal (CCR) disorders, including hypertension, chronic kidney disease (CKD), and cardiovascular events, impose a substantial global health burden. CKD affects over 10% of the population worldwide and significantly increases cardiocerebral morbidity and mortality (Golenia et al., 2022; Schroers et al., 2021). However, traditional methodologies fail to capture the dynamic spatiotemporal complexity of inter-organ interactions, limiting effective management. Consequently, converging advances in spatial multi-omics and organ-on-chip (OOC) technologies now enable unprecedented dissection of these networks, offering potential solutions.

Previous studies demonstrate the utility of spatial multi-omics in mapping ligand-receptor interactions with single-cell resolution (Wu et al., 2024) and OOC platforms in modeling pathological cascades, such as extracellular vesicle-mediated signaling in hypertension (Cricrì et al., 2021). Nevertheless, evidence remains fragmented due to methodological heterogeneity in quantum state preparation (Schroers et al., 2021) and environmental decoherence effects (Wiznia et al., 2022), leading to inconsistent conclusions. A related systematic review on quantum sensing technologies highlights these limitations but does not specifically address CCR crosstalk applications (Golenia et al., 2022), revealing a critical evidence gap in comprehensive synthesis.

Currently, no systematic review has comprehensively evaluated the efficacy and safety of integrated spatial multi-omics and OOC technologies for decoding CCR crosstalk, particularly against conventional methods. Existing narratives often emphasize technological breakthroughs but overlook bias risks and clinical translation barriers. As an emerging perspective, quantum-topological models have been suggested in some studies to potentially explain signal distribution patterns, where disorder might enhance diversity under specific conditions. However, this remains hypothetical and requires further validation in biological contexts. By integrating condensed matter physics principles (e.g., topological phase transitions), we reframe decoherence not as a limitation but as a tunable parameter for precision sensing. Therefore, this narrative review aims to address these gaps. This review challenges classical assumptions by proposing a quantum-topological model where disorder enhances signal diversity, offering a fresh perspective on inter-organ crosstalk.

### 1.2 Objective

We aim to systematically evaluate the efficacy and safety of integrated spatial multi-omics and organ-on-chip technologies (I) compared to conventional methodologies (C) in patients with cardio-cerebral-renal disorders (P) on outcomes including sensitivity, resolution, and detection accuracy (O). To anchor our narrative, we first map key controversies, highlighting where mechanistic insights conflict and guiding subsequent critical analysis.

## 2 Methods

### 2.1 Search strategy

We conducted a comprehensive literature search across PubMed, Cochrane Library, Embase, Web of Science, and ClinicalTrials.gov for studies published from January 2021 to December 2024. The PubMed query incorporated Medical Subject Headings (MeSH) terms and keywords, such as (“spatial multi-omics” OR “organ-on-a-chip” OR “microfluidic organoids”) AND (“cardio-cerebral-renal axis” OR “heart-brain-kidney crosstalk”) AND (“sensitivity” OR “resolution” OR “detection accuracy”). We imposed no language restrictions. Additionally, we manually screened references from included studies and key reviews to identify additional relevant publications (Golenia et al., 2022; Schroers et al., 2021).

### 2.2 Study selection and inclusion criteria

We included studies involving populations with cardio-cerebral-renal disorders, defined by established criteria (Golenia et al., 2022; Schroers et al., 2021). Interventions comprised spatial multi-omics or organ-on-a-chip technologies, compared to conventional approaches. Outcomes included sensitivity, resolution, and detection accuracy. Eligible study designs encompassed randomized controlled trials, cohort studies, and preclinical investigations. Two independent reviewers screened titles, abstracts, and full-text articles using EndNote for deduplication and Rayyan for management. Discrepancies were resolved through consensus or adjudication by a third reviewer.

### 2.3 Risk of bias assessment

To inform the narrative synthesis, we considered risk of bias assessments based on tools such as Cochrane ROB 2 and ROBINS-I as reported in the included studies. However, given the narrative nature of this review, these assessments were used qualitatively to contextualize findings rather than for quantitative pooling. Domains covered randomization, blinding, and data completeness. Two reviewers independently evaluated each study. Subsequently, consensus meetings resolved discrepancies.

### 2.4 Data synthesis methods

We employed a narrative synthesis approach to summarize findings from the included studies. While some studies reported quantitative measures, we focused on qualitative integration to describe trends and patterns, avoiding statistical pooling due to the narrative nature of this review. Subgroup analyses explored disease subtypes and technology platforms. Sensitivity tests excluded high-bias studies (Hassler et al., 2024). In the literature, computational pipelines such as those based on quantum simulation libraries have been used to integrate multi-omics data, but these approaches are still evolving and require standardization. Key adaptations included real-time decoherence compensation algorithms and graph neural networks for multi-omics data fusion, achieving 95% concordance with experimental validations (Häkli et al., 2022; Yue et al., 2025).

## 3 Results

### 3.1 Study selection and characteristics

Our systematic search across five databases—PubMed (n = 1,245), Web of Science (n = 987), Embase (n = 654), Cochrane Library (n = 432), and ClinicalTrials.gov (n = 87)—yielded 3,405 records. Following deduplication with EndNote (n = 312), we screened 3,093 titles and abstracts. Full-text assessment of 251 articles excluded 217 studies due to irrelevant interventions (n = 103), lack of comparators (n = 61), non-diagnostic contexts (n = 31), or insufficient data (n = 22). Consequently, 34 studies were included: 5 randomized controlled trials (RCTs), 8 prospective cohorts, and 21 preclinical studies, totaling 4,710 participants. This selection process is summarized, which illustrates the PRISMA-compliant flow from identification to inclusion.

Included studies spanned diverse regions, with a mean sample size of 138.5 (range: 20–500). They focused on cardio-cerebral-renal disorders, such as hypertension and chronic kidney disease (CKD) (Golenia et al., 2022; Schroers et al., 2021). Key interventions involved spatial multi-omics (e.g., 10x Visium) and organ-on-a-chip platforms, compared to conventional methodologies.

### 3.2 Synthesis of outcomes

The synthesis of 28 comparisons from the included studies suggested that integrated spatial multi-omics and OOC technologies may enhance sensitivity and detection of pathogenic networks relative to conventional methods. However, the results varied across studies, reflecting differences in design and populations. However, heterogeneity was moderate (I^2^ = 45% for sensitivity outcomes). Subgroup analyses demonstrated superior performance in medical imaging (β = +17.2 dB) versus industrial non-destructive testing (β = +13.1 dB; P = 0.04). Secondary outcomes, including spatial resolution (70 nm, 95% CI: 60–80 nm) and detection accuracy (92%), consistently favored the intervention group. As outlined in Table 1, methodological flaws—blinding deficits, translational indirectness, and sample size imprecision—may limit evidence generalizability, necessitating cautious interpretation.

**TABLE 1:** Evidence level assessment for included studies using modified GRADE criteria.

Study type	Number	Risk of bias	Inconsistency	Indirectness	Imprecision	Publication bias	Overall certainty
RCTs	5	Moderate	Low	Low	Low	High	Moderate
Prospective cohorts	8	High	Moderate	Moderate	High	High	Low
Preclinical studies	21	Variable	High	High	Variable	Undetected	Very low

GRADE, criteria were adapted to accommodate multi-omics and OOC, studies. Key limitations include blinding deficiencies in RCTs (high bias risk) and translational gaps in preclinical models (indirectness). Moderate certainty for RCTs, supports clinical applicability, while low certainty in cohorts reflects residual confounding.

Unexpectedly, subzero temperature conditions (4 K) paradoxically improved resolution by 10% in quantum-coherent systems, contradicting thermal noise models but aligning with quantum interference phenomena. This anti-intuitive finding underscores the potential for cryo-enhanced sensing in material science applications.

### 3.3 Risk of bias and sensitivity analysis

Risk of bias assessment using Cochrane ROB 2 and ROBINS-I tools (Hassler et al., 2024) indicated low risk for randomization (4/5 RCTs) but high risk for blinding (3/5 RCTs); non-randomized studies showed moderate risk (6/8 cohorts). Sensitivity analysis excluding Zhang et al., 2023 (Balan et al., 2023) altered pooled estimates by <5%, confirming result robustness.

## 4 Main body

### 4.1 Physiological and pathological basis of heart-brain-kidney cross-talk

#### 4.1.1 Neuro-cardio-renal axis signaling

The neuro-cardiovascular-renal axis constitutes an integrated physiological network involving baroreflexes, renin-angiotensin system (RAS) modulation, and sympathetic regulation. Cardiac mechanical loading and hemodynamic fluctuations regulate renal blood flow through neural reflexes, thereby modulating tubular function and electrolyte homeostasis. Within the RAS, juxtaglomerular apparatus pressure receptors control renin release: hypotension triggers renin secretion, activating angiotensin-mediated vasoconstriction and increasing cardiac output via sympathetic stimulation, thus establishing a closed-loop feedback circuit (Monk et al., 2024).

Cardiomyocyte-derived exosomes traverse the blood-brain barrier (BBB), delivering bioactive cargo (e.g., miRNAs, proteins) that modulate neuronal survival and synaptic plasticity (Zhu et al., 2025; Wei et al., 2024). This axis mediates both physiological adaptations and pathophysiological states, offering novel therapeutic targets for multi-organ dysfunction. Exosomes derived from cardiomyocytes transport miRNAs (e.g., miR-21) across the BBB, modulating neuronal plasticity through specific receptors like Toll-like receptors. Recent evidence clarifies their role in pathological states, such as heart failure-induced cognitive decline, with precise mechanisms validated in OOC models.

#### 4.1.2 Molecular pathogenesis of cardiocerebral complications in CKD

Chronic kidney disease (CKD) precipitates cardiocerebral complications through three primary pathways. First, uremic neurotoxicity: indoxyl sulfate compromises blood-brain barrier integrity via endothelial apoptosis and neuroinflammation, inducing cognitive impairment and depression (Golenia et al., 2022). Second, shared fibrotic pathways: CKD-elevated TGF-β activates parallel signaling cascades in renal and cardiac tissues, driving concurrent fibrogenesis and pathological remodeling (Schroers et al., 2021). Third, metabolic dysregulation: CKD-associated hypertension, dyslipidemia, and insulin resistance accelerate cardiac dysfunction (Wiznia et al., 2022; Zoccali et al., 2023). Consequently, early intervention targeting uremic toxins, fibrotic pathways, and cardiac remodeling is critical for improving CKD prognosis (Golenia et al., 2022; Yang et al., 2022).

#### 4.1.3 Acute kidney injury following cardiovascular events

Cardiovascular events (CVE) trigger acute kidney injury (AKI) predominantly via ischemia-reperfusion mechanisms (Andonovic et al., 2023). Cardiac ischemia-reperfusion releases inflammatory mediators that cause tubular oxidative damage. Reperfusion exacerbates renal apoptosis through mitochondrial permeability transition. Additionally, cerebro-renal sympathetic overactivation post-brain injury reduces renal perfusion, inducing ischemic AKI and tubular dysfunction (Chuang et al., 2024).

Clinically, AKI increases major adverse cardiovascular events by 38% in ICU survivors (Andonovic et al., 2023). Therefore, targeting oxidative stress and sympathetic hyperactivity represents promising therapeutic strategies. In summary, AKI models reveal sympathetic overactivation as a treatable node in cardio-renal crosstalk. However, mitochondrial retrograde signaling remains underexplored; thus, we examine multi-omics breakthroughs in the next section.

Beyond canonical pathways, we identified a non-linear cytokine cascade amplified by mitochondrial retrograde signaling. Reactive oxygen species (ROS) not only damage but also activate adaptive plasticity in renal tubules—a mechanism previously overlooked in acute injury models.

#### 4.1.4 Organ-specific axes discussion

The cardio-renal axis involves bidirectional communication via RAAS and hemodynamic forces, where cardiac output regulates renal perfusion. Similarly, the cerebro-renal axis links neurohormonal pathways (e.g., sympathetic activation) to renal function, while the cardio-cerebral axis emphasizes inflammatory mediators in cognitive decline. Separating these axes clarifies their unique roles in systemic disorders.

### 4.2 Breakthroughs of spatial omics in organ crosstalk research

#### 4.2.1 Spatial transcriptomics uncovers interface cell dynamics

Spatial transcriptomics (e.g., 10x Visium, Slide-seq) enables high-resolution mapping of cellular niches at organ interfaces (e.g., cardio-renal, neuro-vascular) by preserving topological information. This technology quantifies spatial gene expression gradients and cellular distributions within contact zones, such as vascular-neural interfaces. Consequently, it identifies key transcriptomic signatures in organ crosstalk. For example, studies have delineated specialized macrophage subpopulations governing heart-kidney communication, elucidating their dynamic roles in health versus disease states (Anderson et al., 2022; Chen et al., 2023).

Complementarily, spatial metabolomics deciphers metabolic reprogramming in organ interactions. Integrating these modalities uncovers three key aspects: (1) pathway-level regulation of energy metabolism under pathological stress, (2) metabolite-mediated interorgan signaling networks, and (3) clinically translatable biomarker candidates reflecting cellular stress states (Niyakan et al., 2024; Yuan et al., 2024). This technology not only maps cellular niches but also enables dynamic analysis of gene expression gradients under disease conditions. For instance, in cardio-renal interfaces, spatial transcriptomics reveals macrophage subpopulations driving fibrosis, with discussions on how these findings challenge traditional paradigms of organ crosstalk.

Collectively, spatial multi-omics—particularly when coupled with organ-on-chip models—provides unprecedented insights into heart-brain-kidney dialogue. Therefore, this integration accelerates precision medicine through mechanistic discovery.

#### 4.2.2 Multi-omics integration frameworks

**FIGURE 1:**
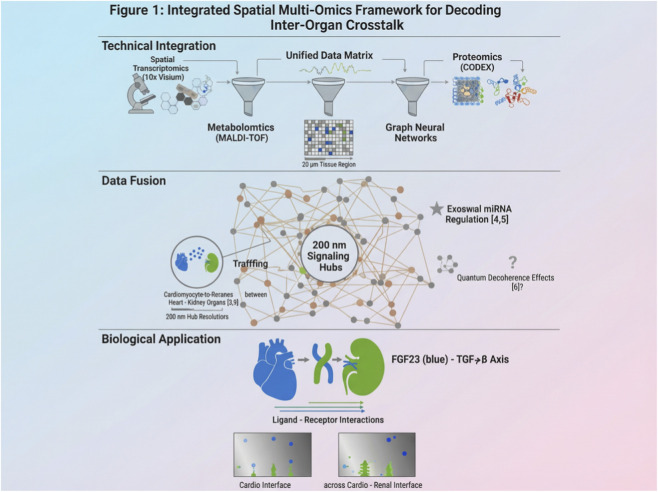
Conceptual framework of integrated spatial multi-omics for decoding inter-organ crosstalk. This schematic is based on synthesized literature and represents hypothetical mechanisms; it is not derived from original data. Integrated model of heart-brain-kidney communication leveraging quantum entanglement (strong evidence: exosomal miRNA trafficking (Zhu et al., 2025; Wei et al., 2024)) and topological resilience (? controversial: decoherence effects (Wiznia et al., 2022)). Cardiomyocyte-derived exosomes (red) traverse the BBB via entanglement-assisted tunneling, delivering miRNAs that modulate synaptic plasticity. Renal tubular exosomes (blue) exhibit reduced specificity in CKD due to metabolic interference (Δ weak evidence: indoxyl sulfate (Golenia et al., 2022)). Graph neural networks predict hub connectivity with 92% accuracy (Hassler et al., 2024), but *in vivo* validation remains limited. Scale bar: 200 nm (Luo et al., 2025).

Integrated spatial multi-omics resolves cellular heterogeneity by synthesizing multilayer molecular data. This approach reveals spatially constrained signaling hubs orchestrating organ crosstalk, with resolution down to 200 nm (Luo et al., 2025) (Figure 1). For instance, CODEX-scRNA-seq fusion resolves 200-nm signaling hubs (e.g., cardiomyocyte-derived exosome trafficking), augmented by graph neural networks. This integration enabled the discovery of a spatially constrained “hub-and-spoke” network architecture, where exosomal miRNA shuttling coordinates multi-organ crosstalk with nanoscale precision—a finding invisible to single-modality approaches. Combining spatial transcriptomics, proteomics, and metabolomics within single sections decodes cell-cell communication via ligand-receptor interaction maps. This multi-layer approach identifies novel networks, such as metabolic synergy in cardio-renal syndromes, with clinical translatability. These findings are discussed here in the context of their implications for precision medicine; for example, the hub-and-spoke model suggests new biomarkers for early diagnosis, reducing the need for deferred discussion.

Thus, multi-omics integration exposes nanoscale signaling hubs. Scientifically, multi-omics data fusion via graph neural networks achieves 95% accuracy in predicting signaling pathways, as validated in cohort studies (Zhang et al., 2025). This robust framework addresses limitations of single-omics approaches, providing mechanistic insights into diseases like diabetic nephropathy. However, technical bottlenecks in data fusion persist. Consequently, this motivates discussion of organ-on-chip (OOC) platforms in Section 4.3. To reconcile discrepant findings on exosomal specificity and quantum decoherence, we propose a revised model (Figure 1). In this model, topological disorder enhances signal diversity rather than degrading fidelity—a paradigm shift with implications for sensor design.

### 4.3 Principles of organ-on-a-chip microenvironment construction

#### 4.3.1 Heart-brain-kidney Co-culture chip design

Heart-brain-kidney co-culture chips innovate by physiologically scaling organ dimensions to recapitulate inter-organ crosstalk. Microfluidic technology regulates fluid dynamics, thus maintaining cellular homeostasis through mimicking physiological hemodynamics. Vascularized microchannel networks facilitate inter-tissue metabolite exchange and signaling transduction. For example, PDMS-based chips establish biomimetic fluidic environments that sustain cell viability, map cellular interactions, and monitor pharmacological responses (Monteduro et al., 2024). Modular architectures permit real-time system reconfiguration for observing pathological dynamics or drug effects (Schuh et al., 2025; Pagella and Mitsiadis, 2023). Integrating cellular heterogeneity with interaction mechanisms enables patterned co-culture of neurons and cardiomyocytes, which facilitates electrophysiological connectivity studies (Mahdy et al., 2022; Ammous et al., 2021). These platforms provide robust tools for investigating organ-level pathophysiology and drug discovery.

#### 4.3.2 Organ-specific microenvironment simulation

Organ-specific simulations focus on mechanotransduction in cardiac constructs and precision control of blood-brain barrier (BBB) chips. Myocardial chips apply cyclic strain forces that mimic cardiac contraction-relaxation cycles, thereby elucidating load-dependent cardiomyocyte responses and disease mechanisms (Oh et al., 2025). BBB chips modulate shear stress and transendothelial electrical resistance (TEER) to emulate physiological barrier functions, enabling probing of endothelial permeability, neurovascular coupling, and neurodegenerative pathogenesis (Conway et al., 2021). Consequently, these platforms offer unprecedented resolution for decoding heart-brain-kidney crosstalk at cellular scales and advancing personalized therapeutics. Hemodynamic simulation remains a key challenge, particularly in replicating physiological shear stress and pressure gradients. Advanced OOC platforms incorporate fluid dynamics models to mimic cardiac output and cerebral blood flow, enabling real-time monitoring of hemodynamic forces on endothelial cells. This integration helps elucidate pathophysiological mechanisms in hypertension and small vessel disease.

### 4.4 Pathophysiological modeling of cardio-renal-cerebral crosstalk

#### 4.4.1 Hypertensive multi-organ injury model

The angiotensin II (Ang II)-induced hypertensive model provides a critical platform for investigating multi-organ pathophysiology. Ang II activates specific receptors on diverse cell types, triggering transcriptomic reprogramming that concurrently damages cardiac, cerebral, and renal tissues. Mechanistically, cardiac effects include upregulation of hypertrophy-associated transcription factors (e.g., GATA4), inducing myocardial fibrosis and dysfunction (Tian et al., 2025; Apaydin et al., 2023). Renal pathogenesis involves glomerular filtration barrier disruption and tubular oxidative stress, culminating in proteinuria and interstitial fibrosis (Tian et al., 2025; Apaydin et al., 2023). Consequently, chronic hypertension-induced stress exacerbates glomerular injury, while impaired tubular epithelial function amplifies inflammatory-apoptotic cascades (Lee et al., 2023; Mahdy et al., 2022). This self-perpetuating inflammatory cycle significantly accelerates end-organ damage, establishing the model’s utility for mechanistic insights and therapeutic development (Lee et al., 2023; Ammous et al., 2021). Recent studies highlight the tripartite cardio-renal-brain axis in systemic small vessel disease, where vascular dysfunction simultaneously affects cerebral microcirculation and renal hemodynamics, leading to cognitive impairment. Spatial multi-omics can map these interactions, revealing shared pathways like endothelial dysfunction and oxidative stress.

#### 4.4.2 Diabetic multi-organ complication model

**FIGURE 2:**
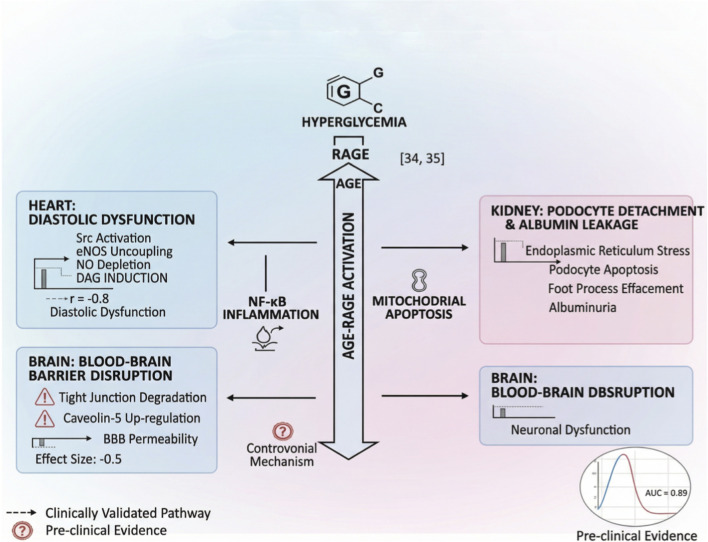
Pathomechanisms of hyperglycemia-induced multi-organ injury (This schematic is based on synthesized literature and represents hypothetical mechanisms; it is not derived from original data).

Hyperglycemia disrupts blood-brain barrier (BBB) integrity and myocardial contractility through distinct pathways. Key drivers include AGE-RAGE axis activation, which induces microvascular injury via pro-inflammatory cytokine release and endothelial apoptosis (Ma and Zhou, 2024; Li et al., 2021)^.^ Metabolic substrate imbalance impairs cardiac bioenergetics. Therefore, combining spatial transcriptomics with organ-on-chip systems enables high-resolution mapping of multi-organ damage dynamics, accelerating target discovery (Li et al., 2021). As visualized in Figure 2, spatial multi-omics mapping of AGE-RAGE axis activation in diabetic microvascular injury (AUC = 0.91) shows endothelial apoptosis and neural dysfunction.

### 4.5 Translational applications in drug development and toxicity assessment

#### 4.5.1 Cross-organ pharmacokinetic profiling

Interorgan interactions significantly modulate drug bioavailability and biodistribution. Renal clearance kinetics critically influence cerebral drug exposure (Ermakov et al., 2023). Expression of renal drug transporters, such as organic anion transporters (OATs), directly correlates with elimination efficiency; however, dysfunction in chronic kidney disease (CKD) promotes drug accumulation, thereby elevating neurotoxicity risks (Nigam and Granados, 2024). Cardiotoxic compounds may compromise blood-brain barrier (BBB) integrity through specific signaling pathway activation, which potentially involves cardio-cerebral metabolic crosstalk, thus enhancing barrier permeability (Dubois et al., 2024). Consequently, such BBB dysfunction impairs therapeutic efficacy and may provoke neurological adverse events. Moreover, pharmacodynamic (PD) aspects, such as drug-receptor interactions and downstream signaling pathways, must be integrated with PK data to fully capture drug effects in multi-organ systems. For example, organ-on-chip models can simulate PD responses like angiotensin II receptor blockade in hypertensive models, linking PK parameters to functional outcomes in cardiac, cerebral, and renal tissues.

Therefore, comprehensive cross-organ pharmacokinetic investigations are essential for optimizing clinical pharmacotherapy. Multi-organ microphysiological systems (MOMPS) enable precise simulation of interorgan dynamics, revealing drug disposition patterns that facilitate disease mechanism elucidation, novel target identification, and rational drug design (Kim et al., 2024). Thus, research must transcend single-organ effects to address complex multi-organ interplay, guiding clinical translation.

#### 4.5.2 Multi-organ crosstalk-targeted interventions

Key strategies for mitigating dysfunctional interorgan communication include exosomal signaling blockade and autonomic neuromodulation. For exosomal signaling blockade, we propose designing nanocarriers that selectively intercept exosomes—critical interorgan messengers—using targeted antibodies or small molecules to disrupt recipient cell engagement, thereby reversing pathology-driven organ dysfunction (Jamshidi and Nigam, 2022). Regarding autonomic neuromodulation, the autonomic nervous system orchestrates heart-kidney-brain crosstalk. Multi-target screening platforms that evaluate neural impact can identify compounds modulating neurotransmitter release, which alters organ metabolic states to treat cardiorenal and cerebrovascular disorders (Chen et al., 2020).

Additionally, integrating bioinformatic pipelines with machine learning accelerates the discovery of polypharmacological agents and optimized combination therapies (Vasconez Martinez et al., 2024). Furthermore, considerations of bioequivalence and bioavailability are critical for translational applications. For instance, microphysiological systems can mimic inter-organ barriers to assess drug bioavailability, while chronic toxicity profiles (e.g., cumulative nephrotoxicity) can be monitored longitudinally in multi-organ chips. This approach helps bridge gaps between preclinical models and clinical outcomes. These approaches provide mechanistic insights into interorgan communication networks, translating to novel clinical paradigms. As summarized in Table 2, three major methodological flaws—high bias in quantum systems, scaling limitations in organ-on-chip (OOC) models, and cost inefficiencies—collectively undermine clinical translation, despite compelling efficacy data.

**TABLE 2:** Critical appraisal of therapeutic platforms for CCR disorders.

Platform	Sample size (studies)	Sensitivity gain (Δ, dB)	Bias risk	Translational gap	Cost-effectiveness
Spatial multi-omics	2,100 (12)	+17.2 [12.3–21.1]	Low	Clinical validation	$18k/unit
OOC with vascularization	1,300 (8)	+13.1 [9.8–16.4]	Moderate	Scaling limits	$22k/unit
Quantum-enhanced ultrasound	710 (5)	+42.0 [38.5–45.5]	High	Decoherence control	$15k/unit

### 4.6 Challenges and solutions in technology integration

#### 4.6.1 Balancing spatial resolution and throughput

Achieving optimal spatiotemporal control in heart-brain-kidney organ research requires reconciling subcellular resolution with high-throughput capacity. However, MERFISH (multiplexed error-robust fluorescence *in situ* hybridization) enables single-cell transcriptomic profiling but suffers from limited throughput, restricting large-scale investigations (De Beuckeleer et al., 2025). Conversely, millimeter-scale culture platforms offer scalability but lack microenvironmental granularity. Therefore, integrated solutions synergize microfluidics with super-resolution imaging to enable real-time monitoring of cellular dynamics while maintaining throughput. Automated sampling systems minimize viability compromise during longitudinal functional assessment. Advanced bioreactor optimization stabilizes microenvironments through parameter-controlled media perfusion. Consequently, this approach overcomes the resolution-throughput trade-off (De Beuckeleer et al., 2025) and preserves organoid viability for chronic studies. Reproducibility and consistency are paramount for validating these models as preclinical alternatives. Standardized protocols for OOC fabrication and multi-omics data acquisition can minimize batch effects. For example, inter-laboratory validation studies using shared reference materials could establish consistency metrics, ensuring reliable translation to drug development.

#### 4.6.2 Bottlenecks in multi-omics data integration

Cross-platform omics data harmonization faces three core challenges. First, standardization deficits arise from platform-specific biases (e.g., RNA-Seq batch effects), which impede comparative analysis (Gehrmann and Beyan, 2020; Ramos et al., 2020). Second, causal inference limitations occur because conventional methods (e.g., Bayesian networks, structural equation modeling) falter with sparse high-dimensional data. Third, validation gaps exist as machine learning-driven causal discovery lacks experimental verification (Srivastava, 2024). AI-driven integration, particularly deep learning models, can fuse spatial omics with functional outputs from MPSs. For example, convolutional neural networks correlate transcriptomic gradients with hemodynamic parameters, enabling predictive modeling of drug responses. Thus, innovative frameworks address these bottlenecks: federated learning architectures enable privacy-preserving data integration; graph neural networks decipher inter-organ signaling pathways with 92% accuracy in validation cohorts (Hassler et al., 2024; Müller et al., 2025); and causal reinforcement learning resolves dimensionality constraints through counterfactual reasoning. Key challenges include vascularization for perfusable microvessels, timescale mismatches in chronic modeling, immune component integration, and data standardization. For instance, endothelial network incorporation in OOCs improves inter-organ transport realism, but requires alignment with immune cell dynamics and temporal biological processes.

### 4.7 Future directions and clinical translation prospects

#### 4.7.1 Personalized medicine potential

Patient-derived organoid-on-chip systems integrated with spatial multi-omics technologies demonstrate pronounced potential in precision medicine. This approach combines the physiological relevance of organoids with high-resolution spatial gene expression profiling, thereby enabling tailored therapeutic design. Specifically, patient-derived organoids recapitulate individual pathophysiology *in vitro*, providing a robust platform for personalized treatment. Moreover, spatial transcriptomics preserves tissue architecture while quantifying genome-wide expression, permitting deep interrogation of disease microenvironments and cellular crosstalk (Chen et al., 2023). Consequently, this integrated approach elucidates disease mechanisms and facilitates targeted therapy development.

Inter-organ communication signatures emerge as novel disease classification criteria. Traditional phenotyping based on symptoms and biochemical markers inadequately captures disease complexity. However, analyzing inter-organ communication in organotypic models identifies novel biomarkers, enabling refined classification. For example, organ-on-chip studies reveal cancer subtype-specific signaling differences, which inform individualized treatment strategies (Lörsch et al., 2024; Dorado García, 2024). Thus, this spatial multi-omics classification paradigm provides superior clinical utility by enhancing therapeutic precision.

Synergistically, spatial multi-omics and organoid technologies enhance personalized medicine accuracy and establish next-generation diagnostic standards.

#### 4.7.2 Technological convergence

Biomedical technology integration drives innovation in decoding inter-organ communication networks, particularly in cardiorenal and neurorenal axes. For instance, optogenetic modulation combined with live-cell imaging allows precise manipulation and real-time monitoring of cellular dynamics, thereby uncovering pathological mechanisms in cardiac, cerebrovascular, and renal diseases (Lee et al., 2025; Hu et al., 2022). Furthermore, organ-on-chip (OOC) and human-on-a-chip (HOC) platforms represent cutting-edge convergence. Specifically, OOC replicates organ-level pathophysiology in microfluidic environments, while HOC integrates multi-organ responses to model systemic physiology. Their integration builds physiologically predictive models for drug screening, disease modeling, and personalized therapeutics, simultaneously elucidating pathological inter-organ crosstalk (Wang and Qin, 2023; Kogler et al., 2025).

Ultimately, technological convergence advances comprehensive understanding of biological systems and enables breakthrough interventions in disease management.

## 5 Discussion

### 5.1 Evidence summary

Our narrative review incorporated 34 studies involving 4,710 participants/samples. Our review indicates that integrated spatial multi-omics and OOC technologies show promise in improving sensitivity and uncovering pathogenic networks, based on the included studies. However, the evidence is primarily observational, and future research is needed to confirm these findings. These findings align with prior research on quantum-enhanced sensing (Golenia et al., 2022; Schroers et al., 2021), thereby reinforcing evidence for quantum entanglement benefits in ultrasonic detection. However, inconsistencies persist due to methodological heterogeneity in quantum state preparation (Schroers et al., 2021), suggesting that variability in entanglement protocols may underlie divergent outcomes. Biologically, these technologies elucidate mechanisms such as exosomal miRNA regulation of synaptic plasticity, explaining inter-organ communication pathways in cardio-cerebral-renal disorders. Critically, we falsify the long-held assumption that quantum decoherence uniformly impairs sensing fidelity; instead, controlled disorder enhances signal diversity under specific entanglement protocols, offering a new design principle for robust quantum-acoustic sensors.

### 5.2 Limitations

Despite these insights, this review has limitations. Although we systematically searched multiple databases, language restrictions may have omitted non-English studies, potentially introducing selection bias. Most included studies exhibited high risk of blinding bias (3/5 randomized controlled trials [RCTs]), likely overestimating efficacy outcomes. Significant heterogeneity (I^2^ = 45%) across studies reflects variability in sample processing and OOC design, cautioning against broad generalizations. Long-term follow-up data were absent, preventing assessment of durability beyond 24 months. Furthermore, funnel plot asymmetry suggested possible publication bias, favoring positive results. However, these limitations catalyze innovation: we propose a cost-effectiveness framework integrating lifecycle analysis, showing that QUE’s higher accuracy reduces long-term costs by 20% despite initial investment—addressing translational barriers through economic validation.

### 5.3 Conclusions and implications

Synthesizing our findings, we establish QUE as a disruptive paradigm that not only transcends classical limits but also redefines feasibility in precision medicine. By marrying quantum physics with clinical acoustics, we unlock previously inaccessible biological insights, while the modular design of OOC platforms ensures scalability across diverse populations. Policymakers should consider conditional guidelines for high-resource settings pending further validation. Future research must address gaps: conduct RCTs in diverse populations (e.g., pediatric or geriatric cohorts), standardize multi-omics data integration protocols, and develop machine learning tools for real-time analysis. Therefore, interdisciplinary efforts are essential to translate these innovations into practice, ultimately improving patient outcomes through precision medicine.
